# Identification of a Cardiac Myxoma on a Breast MRI

**DOI:** 10.1016/j.jaccas.2025.105431

**Published:** 2025-09-16

**Authors:** Nolan Shoukri, Matthew Hanna, Nishaki Mehta, Cristian Chagas, Lauren Stein, Michael Gallagher

**Affiliations:** a Oakland University William Beaumont School of Medicine, Rochester, Minnesota, USA; b Cardiovascular Medicine, Corewell Health William Beaumont University Hospital, Royal Oak, Michigan, USA; c Diagnostic Radiology, Corewell Health William Beaumont University Hospital, Royal Oak, Michigan, USA

**Keywords:** cardiac tumor, left atrium, myxoma, screening MRI

## Abstract

**Background:**

Cardiac myxomas are a rare, benign, and often asymptomatic tumor, commonly found in the left atrium. They are typically detected incidentally via echocardiography or other imaging modalities and are usually treated with surgical resection.

**Case Summary:**

A 44-year-old woman presented to our hospital due to a left atrial mass incidentally identified on a screening breast magnetic resonance imaging. Follow-up echocardiogram, computed tomography, and cardiac magnetic resonance confirmed the diagnosis of cardiac myxoma with thrombus formation.

**Discussion:**

The patient was asymptomatic; however, her family history of early onset breast cancer prompted vigilant screening, leading to incidental detection of the mass. Careful review by an observant radiologist facilitated early recognition and timely intervention.

**Take-Home Message:**

As imaging becomes more widespread and sophisticated, multimodality training could allow for early identification and treatment of many significant findings such as this myxoma, preventing adverse outcomes.

## Graphical Abstract

**Figure:**
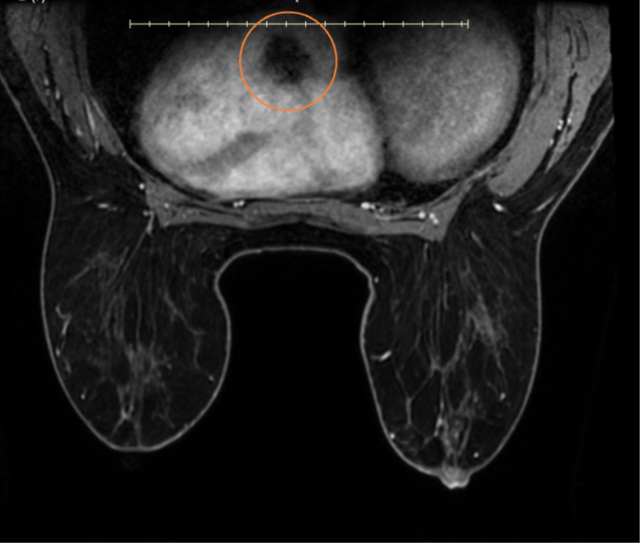

Primary cardiac tumors are extremely rare, appearing in <1% of autopsies. Among these, approximately 75% are benign.^1^^,^^2^ In contrast, most cardiac tumors are metastatic in origin. When present, cardiac tumors are often asymptomatic and identified incidentally. When symptoms do arise, they are typically related to the tumor's size, location, and its impact on normal tissue function. Imaging modalities, including echocardiography, computed tomography (CT), and cardiac magnetic resonance (cMRI), are commonly used for diagnosis, whereas positron emission tomography imaging can help differentiate benign from malignant tumors.^1^^,^^2^ A literature review of 50 recent articles related to cardiac myxoma showed that echocardiography was the most common test used to identify cardiac myxomas (71.4%), followed by CT (25.7%), and cMRI (2.9%).

**Take-Home Messages**

• Cardiac myxomas, although benign, can pose a significant threat to the health of the patient either through physical interruption of cardiac flow or thrombus formation.
• As imaging modalities and screening imaging become more commonplace, advanced multimodality training is essential to ensure that incidental findings are not overlooked.

Cardiac myxomas are the most common primary cardiac tumor. They typically affect adult women and most often arise in the left atrium. They generally have a round/oval polypoid shape and are often isolated in their formation. Systemic embolism is a recognized complication of cardiac myxoma, with its clinical impact varying based on the destination of the embolism. This occurs in approximately 1 in 3 patients and may present as myocardial infarction, cerebrovascular event, or peripheral embolism.^1^^,^^3^ Most cardiac myxomas are sporadic; however, a small proportion are associated with Carney complex, a rare autosomal dominant disorder characterized by recurrent and multiple cardiac myxomas, spotty skin pigmentation, and endocrine tumors. Cardiac myxomas are often identified when they are on average 5 to 6 cm in size and can grow as much as 0.5 cm/mo.^4^^,^^5^ The first-line treatment of cardiac myxomas is surgical removal due to the risk of embolization and the pedunculated nature of the tumor.^1^, ^2^, ^3^

## Presentation

A 44-year-old asymptomatic woman with a family history of early onset breast cancer was undergoing guideline-directed high-risk breast cancer surveillance with alternating screening mammography and breast magnetic resonance imaging (MRI). Her screening breast MRI revealed a filling defect within the left atrium. Dedicated cardiac imaging was recommended, prompting her presentation to the emergency department. The patient denied chest pain, shortness of breath, syncope, dizziness, sweating, fever, chills, and malaise. She reports occasional palpitations related to a prior diagnosis of premature ventricular contractions. Previous cardiac imaging studies were unremarkable, including an echocardiogram (5 years prior) and CT calcium scoring (2 years prior).

## Past medical history

Her past medical history is notable for hypertension and premature ventricular contractions. She was originally on tamoxifen for breast cancer risk. Family history included early onset coronary artery disease, including myocardial infarction in her father and paternal uncles.

## Differential Diagnosis

The differential diagnosis for a left atrial mass includes primary cardiac tumors, metastatic tumors, and thrombi.^6^ Primary cardiac tumors to consider are rhabdomyoma (typically in younger patients), lipoma, myxoma, fibroma, or sarcoma. Metastatic tumors, which are more common than primary tumors, often arise from melanomas, lung cancer, or breast cancer.^7^

## Laboratory and imaging (investigations)

**Figure 1:**
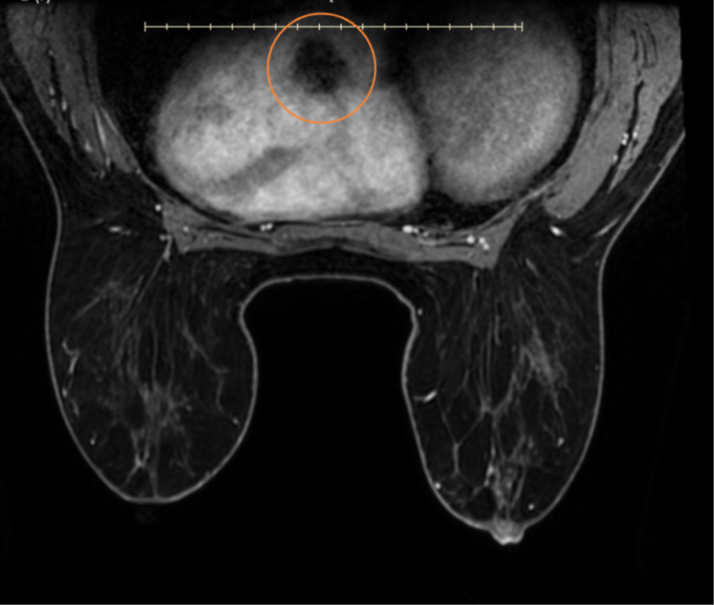
T1-Weighted Postcontrast Magnetic Resonance Imaging Breast magnetic resonance imaging: postcontrast T1-weighted gradient echo fat saturation sequence demonstrates a filling defect within the left atrium (orange circle), suggestive of a cardiac mass.

**Figure 2:**
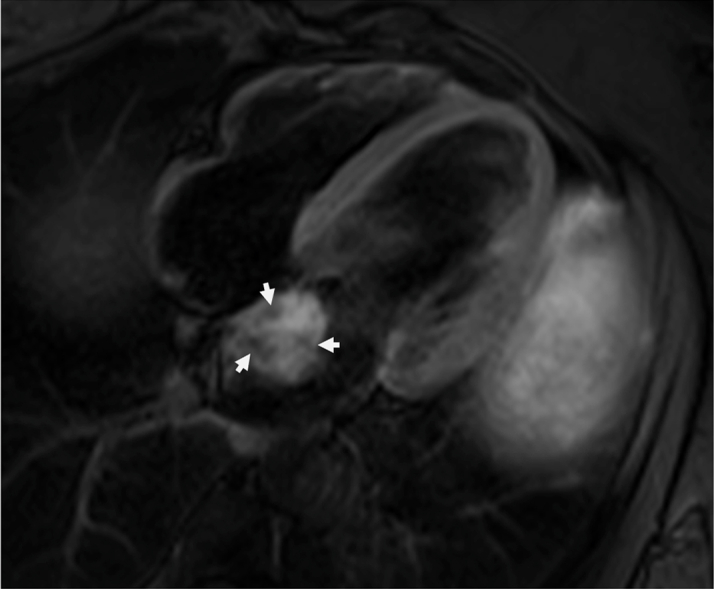
T2-Weighted Cardiac Magnetic Resonance The mass is hyperintense to the myocardium on T2-weighted images. The thrombus is labeled with open arrows, which appears hypointense on T2 imaging compared with the rest of the myxoma.

**Figure 3:**
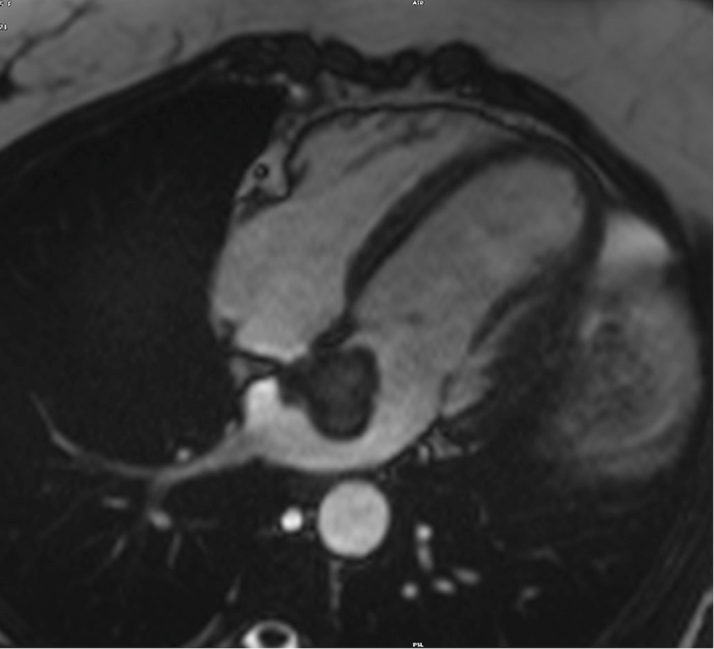
Cardiac Magnetic Resonance 4-Chamber View 4-chamber steady-state free procession imaging: lower signal than the surrounding blood pool; attachment to the interatrial septum.

**Figure 4:**
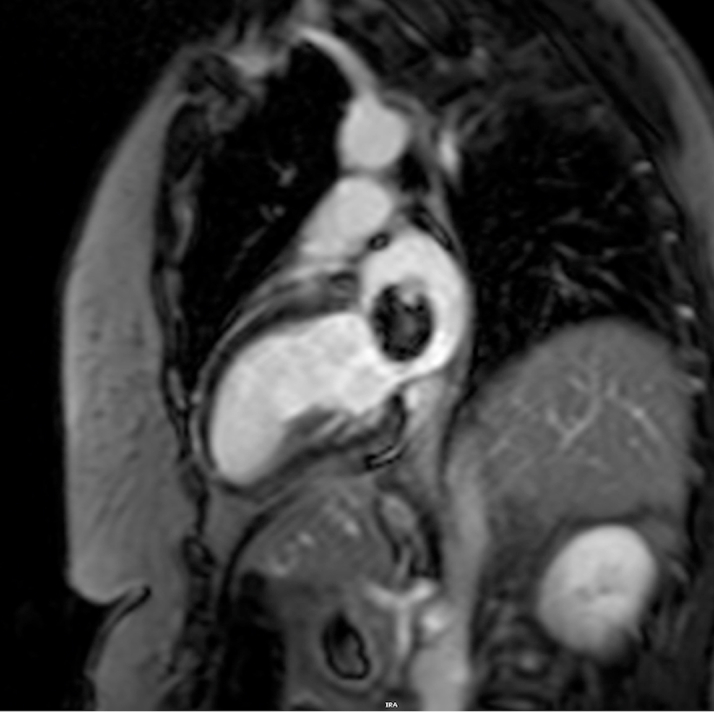
Delayed Enhancement Cardiac Magnetic Resonance Delayed enhancement imaging with heterogenous late gadolinium enhancement of the mass.

**Figure 5:**
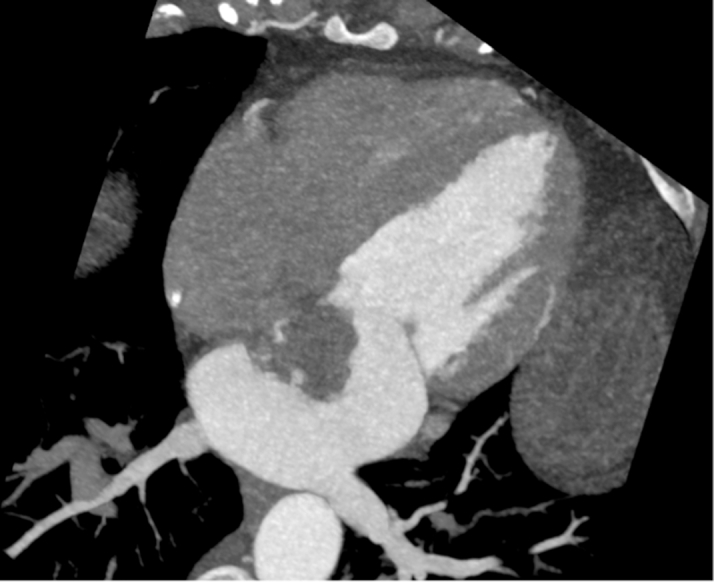
Cardiac Computed Tomography Left atrial myxoma with attachment to the interatrial septum; ovoid with lobulated surface, broad base, appears as an intracavity filling defect in the left atrium; no evidence of calcification.

**Figure 6:**
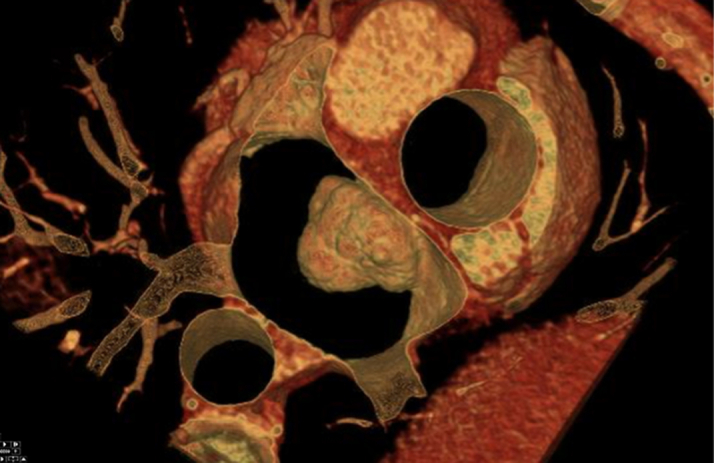
Cardiac Computed Tomography–Derived 3-Dimensional Image Modified surgeons view demonstrating the attachment to the interatrial septum and relationship to the surrounding left atrial anatomy.

The patient's laboratory results were unremarkable. Her complete blood cell count showed a hemoglobin of 13.8 g/dL, high-sensitivity troponin of <4 ng/L, magnesium of 2.2 mg/dL, and international normalized ratio of 1.1. The electrocardiogram showed a normal sinus rhythm. On breast MRI, postcontrast sequences demonstrated a filling defect suggestive of a mass within the left atrium (Figure 1). On admission, the patient underwent a cMRI and cardiac CT. The cMRI demonstrated a left atrial mass fused with the interatrial septum (fossa ovalis) with the following characteristics: hyperintense on T2 (Figure 2), low signal on steady-state free procession imaging (Figure 3), and heterogenous uptake on late enhancement images (Figure 4), all consistent with cardiac myxoma. Cardiac MRI also showed a significant thrombus burden associated with the mass; thrombus associated with the atrial myxoma appears as a region of low or intermediate signal intensity on T2-weighted cMRI. This contrasts with the typically higher and more heterogeneous T2 signal of myxoma tissue itself—the key feature is that thrombus is less hyperintense than tumor tissue on T2-weighted images (Figure 2). An electrocardiogram-gated cardiac CT demonstrated no coronary artery disease and a left atrial myxoma attached to the interatrial septum that is ovoid with a lobulated surface and a broad base (Figure 5), and a 3-dimensional image was derived from the CT (Figure 6).

## Management

The patient underwent minimal right thoracotomy for the complete removal of the left atrial mass. The entirety of the mass was removed with margins. The interatrial septum was reconstructed with a bovine patch. Removal was overall uncomplicated with extubation in the operating room and removal of the chest tube on postoperative day 1.

## Outcome and follow-up

The patient recovered well and was discharged on hospital day 8 (postoperative day 3) with only incisional pain. She is expected to be homebound for 2 to 4 weeks. Follow-up outpatient showed adequate wound healing with no chest pain or shortness of breath, and no hematoma at her surgical site.

## Discussion

This case highlights the incidental detection of a large, asymptomatic cardiac myxoma during screening breast MRI, with subsequent removal, avoiding potential catastrophic complications. Breast MRIs have increased by >55% in utility over the past few decades, along with the rise of other imaging modalities, making incidental findings such as this case much more likely. After our patient was admitted, echocardiogram (Videos 1 and 2) and cMRI assisted in confirming the diagnosis while also identifying concerning thrombus formation on the tumor. The mass was surgically removed, and the patient recovered well without any significant complications.

Despite being asymptomatic, the myxoma was appropriately removed. The patient was young and healthy and did not have any contraindication to surgery. There are no evidence-based nonsurgical managements for cardiac myxomas, and with the risk of future mechanical stress and embolic risk, removal of the myxoma was warranted. The only contraindication to surgery would be recent stroke or intracerebral hemorrhage, and prompt surgery is warranted in cases of villous myxomas due to even greater embolic risk which can be up to 30%.^2^^,^^8^

## Conclusions

Although cardiac myxomas are often asymptomatic, they carry a significant risk of embolic events. Imaging remains central to their identification and diagnosis. As imaging technologies advance and screening becomes more common, it can potentially be used as a screening method. This case highlights the importance of multidisciplinary awareness and interpretation because the astute radiologist identified and brought notice to the left atrial mass incidentally seen on our patient's breast MRI. Maintaining multidisciplinary knowledge rather than focusing on individual parts of the patient allows us to better treat the patient.

## Funding Support and Author Disclosures

The authors have reported that they have no relationships relevant to the contents of this paper to disclose.

## Appendix

Video 1 Video of 4-Chamber View Echocardiogram Without Color Highlighting the Cardiac Myxoma.

Video 2 Video of 4-Chamber View Echocardiogram With Color Highlighting the Cardiac Myxoma and Surrounding Flow Demonstrating Lack of Hemodynamic Compromise.
